# An intraductal human-in-mouse transplantation model mimics the subtypes of ductal carcinoma *in situ*

**DOI:** 10.1186/bcr2358

**Published:** 2009-09-07

**Authors:** Fariba Behbod, Frances S Kittrell, Heather LaMarca, David Edwards, Sofia Kerbawy, Jessica C Heestand, Evelin Young, Purna Mukhopadhyay, Hung-Wen Yeh, D Craig Allred, Min Hu, Kornelia Polyak, Jeffrey M Rosen, Daniel Medina

**Affiliations:** 1 Department of Pathology and Laboratory Medicine, The University of Kansas Medical Center, 3901 Rainbow Blvd., Kansas City, KS 66160, USA; 2 Department of Molecular and Cellular Biology, Baylor College of Medicine, One Baylor Plaza, Houston, TX 77030, USA; 3 Department of Biostatistics, The University of Kansas Medical Center, 3901 Rainbow Blvd., Kansas City, KS 66160, USA; 4 Department of Pathology and Immunology, Washington University School of Medicine, 660 S. Euclid Ave., Campus Box 8118, St. Louis, MO 63110, USA; 5 Department of Medical Oncology, Dana-Farber Cancer Institute and Harvard Medical School, 44 Binney Street, Dana 740C, Boston, MA 02115, USA

## Abstract

**Introduction:**

Human models of noninvasive breast tumors are limited, and the existing *in vivo *models do not mimic inter- and intratumoral heterogeneity. Ductal carcinoma *in situ *(DCIS) is the most common type (80%) of noninvasive breast lesions. The aim of this study was to develop an *in vivo *model whereby the natural progression of human DCIS might be reproduced and studied. To accomplish this goal, the intraductal human-in-mouse (HIM) transplantation model was developed. The resulting models, which mimicked some of the diversity of human noninvasive breast cancers *in vivo*, were used to show whether subtypes of human DCIS might contain distinct subpopulations of tumor-initiating cells.

**Methods:**

The intraductal models were established by injection of human DCIS cell lines (MCF10DCIS.COM and SUM-225), as well as cells derived from a primary human DCIS (FSK-H7), directly into the primary mouse mammary ducts *via *cleaved nipple. Six to eight weeks after injections, whole-mount, hematoxylin and eosin, and immunofluorescence staining were performed to evaluate the type and extent of growth of the DCIS-like lesions. To identify tumor-initiating cells, putative human breast stem/progenitor subpopulations were sorted from MCF10DCIS.COM and SUM-225 with flow cytometry, and their *in vivo *growth fractions were compared with the Fisher's Exact test.

**Results:**

Human DCIS cells initially grew within the mammary ducts, followed by progression to invasion in some cases into the stroma. The lesions were histologically almost identical to those of clinical human DCIS. This method was successful for growing DCIS cell lines (MCF10DCIS.COM and SUM-225) as well as a primary human DCIS (FSK-H7). MCF10DCIS.COM represented a basal-like DCIS model, whereas SUM-225 and FSK-H7 cells were models for HER-2^+ ^DCIS. With this approach, we showed that various subtypes of human DCIS appeared to contain distinct subpopulations of tumor-initiating cells.

**Conclusions:**

The intraductal HIM transplantation model provides an invaluable tool that mimics human breast heterogeneity at the noninvasive stages and allows the study of the distinct molecular and cellular mechanisms of breast cancer progression.

## Introduction

More than 182,000 women were diagnosed with breast cancer, and more than 40,000 died of their disease in 2008 in the United States alone [1]. The 5-year survival rates for noninvasive and locally invasive breast cancers are 98% and 83.3%, respectively. However, the 5-year survival rate is significantly reduced to 27.1% for cancers that have spread to distant sites [1]. Therefore, more research is needed on early detection and prevention, and progress in this field requires a better understanding of the heterogeneity of early breast cancer lesions. Individualized preventive strategies may be the key for effective prevention and ultimately for improved patient survival.

Most DCIS lesions in mice originate from the terminal branches of the mammary tree. This also is true in humans, where the majority of DCIS originate in the terminal duct lobular units (TDLUs). The origin of lobular carcinoma *in situ *(LCIS) is less clear, but most investigators favor the idea that LCIS also originates in the TDLUs. Microarray and histologic studies indicate that the heterogeneity in human breast cancers arises early at the noninvasive stages in DCIS [2]. The mechanisms for invasive progression may be more complex than is reflected in the current histologic and genetic studies. Thus, it becomes important to develop *in vivo *models of human DCIS to investigate the mechanisms involved in the generation of inter- and intralesion heterogeneity. The degree of heterogeneity may explain differences in patient outcome with respect to a risk for malignant progression.

Human breast noninvasive models are limited. Furthermore, the existing models do not mimic the heterogeneity of human disease. To date, two cell-line models are used for the study of noninvasive breast cancers, SUM-225 and DCIS.COM [3]. DCIS.COM was derived from cell culture of a lesion formed by xenotransplantation of MCF10AT cells. Subcutaneous injection of DCIS.COM into nude mice results in rapidly growing lesions that are predominantly comedo (a more-aggressive type of DCIS with central necrosis) [4]. The DCIS lesions appear in about 3 weeks and are composed of luminal epithelial cells surrounded by both a myoepithelial cell layer and a basement membrane. Some areas of early lesions will progress to invasive cancer in about 5 to 6 weeks [5]. SUM-225 was isolated from a chest-wall recurrence of a human DCIS. Similar to those of DCIS.COM, xenografts of the SUM-225 cell line result in the formation of DCIS-like tumors in about 2 weeks. Human DCIS tissue fragments also have been implanted subcutaneously in nude mice with a take rate of about 66% [6].

The aim of the current study was to develop models that would better represent the distinct subtypes of DCIS. We used an intraductal-transplantation model. This method has been used for the delivery of hormones, cytokines, and growth factors to goats, cows, and rats [7]. However, to our knowledge, no previously published literature exists on the use of this method for the purpose of growth and expansion of any breast cancer cell lines or primary cancer cells or for studying the mechanisms of breast cancer invasion. The unique feature of this approach is that the cancer cells are introduced directly into the primary mammary ducts of immunocompromised mice, thus mimicking DCIS in its normal environment. Furthermore, the model allows us to follow the natural progression of human breast cancers (*i.e*., their initial growth as carcinoma *in situ *inside the ducts, sometimes followed by invasion into the stroma by overcoming the barriers of an intact myoepithelial cell layer and a basement membrane. We developed two stable subtype-specific xenograft models, representing a basal subtype and a Her-2-overexpressing subtype. These xenograft lines were developed by using the previously described DCIS-derived cell lines, MCF10-DCIS.COM (here referred to as DCIS.COM) and SUM-225. Additionally, a xenograft line was developed from a human primary DCIS (FSK-H7). Our ultimate goal is to develop multiple models of human primary DCIS to mimic the heterogeneity of human noninvasive breast lesions. Such models should facilitate the design of therapeutic strategies for prevention based on a better understanding of distinct mechanisms of the malignant progression of breast cancer.

## Materials and methods

### Intraductal transplantation method

Recipients are 6- to 10-week-old virgin female SCID-beige mice. Before transplantation, cells are resuspended as single cells in PBS and counted from DCIS.COM, SUM225, or primary human DCIS cells. A 30-gauge Hamilton syringe, 50-μl capacity, with a blunt-ended 1/2-inch needle is used to deliver the cells. The mice are anesthetized, and a Y-incision is made on the abdomen to allow the skin covering the inguinal mammary fat pads to be peeled back to expose the inguinal gland. The nipple of the inguinal gland is snipped so that the needle can be directly inserted through the nipple. Two microliters of cell-culture medium (with 0.1% trypan blue) containing cells at a concentration of 2,500 to 5,000 cells/μl are injected; the injected liquid can be visually detected in the duct. The skin flaps are repositioned normally and held together with wound clips. The primary human DCIS was chopped very finely by using a Teflon block and razor blade or scalpel followed by overnight enzymatic digestion in DMEM/F12 with antibiotics, supplemented with collagenase (1.0 mg/ml) and hyaluronidase (100 U/ml).

Animal and human experiments were conducted by following protocols approved by the Baylor College of Medicine Animal Care and Use and Human Subjects Committee. An informed consent was deemed not to be required by the Human Subjects Committee. [See Additional data file 1 for a video demonstration of the intraductal method.]

### Cell lines

MCF10DCIS.COM and SUM-225 were purchased from Asterand, Inc. (Detroit, MI) and were maintained according to the supplier's guidelines.

### Histologic procedures

Whole mount and H&E were performed as described previously [8].

### Immunofluorescence and reagents

Immunofluorescence (IF) was performed after tissue deparaffinization by clearance in xylene and hydration through graded ethanol series. Microwave antigen retrieval (20 min) in 10 m *M *sodium citrate was performed for all the antibodies used for IF. A 5% solution of bovine serum albumin in phosphate-buffered saline + 0.5% Tween 20 was used as blocking buffer. Sections were incubated with the following primary antibodies overnight at 4°C: ERα rabbit polyclonal 1:50 (Novocastra; 6F11, NCL-ER-6F11, Newcastle Upon Tyne, Tyne and Wear, UK), anti-human SMA 1:100 (1A4-Dako, M0851, Dako, Glostrup, Denmark), anti-mouse SMA 1:200 (A14; Sigma, A2547, St. Louis, MO, USA), CK5 1:50 (XM26, Vector, VP-C400, Burlingame, California, USA), CK8 1:50 (C51, Zymed, 18-0185, San Francisco, California, USA), Her-2 1:50 (SP3, Labvision, RM-9103-SO, Fermont, California, USA), Cytokeratin (AE1/3)1:50 (AE1/AE3, Dako, M3515, Glostrup, Denmark), CK-19 1:50 (Clone A53-B, Lab Vision, MS-198-P0, Fremont, California, USA), and Her-1 1:50 (Clone 31G7, Zymed Laboratories, San Francisco, California, USA). Nuclei were counterstained with 4',6-diamidino-2-phenylindole (DAPI; Vector Laboratories, Burlingame, California, USA) and TO-PRO-3 iodide (Invitrogen, T3605, Carlsband, California, USA). Secondary antibodies included anti-mouse Alexa 488 and anti-rabbit Alexa 594 (Molecular Probes, Carlsband, California, USA). Confocal microscopy was performed by using a laser-scanning confocal microscope (model 510; Carl Zeiss MicroImaging, Inc., Thornwood, NY, USA). The acquisition software used was LSM image browser (Carl Zeiss MicroImaging, Inc., Thornwood, NY, USA). Phase-contrast images were captured with an inverted microscope (CK40-SLP; Olympus). The acquisition software used was Photoshop 5.0 (Adobe). [See Additional data file 2 for detailed information on primary antibodies.]

### Flow cytometry

Primary antibodies used were anti-human MUC-1 1:250 (Stem Cell Technologies, 01423, Vancouver, BC, Canada), CD10-APC 1:10 (BD Bioscience, 340923, San Jose, California, USA), EPCAM-FITC (Stem Cell Technologies, 10109, Vancouver, BC, Canada), Biotinylated AC133 1:5 (Miltenyi Biotec, 130-090-664, Bergisch Gladbach, Germany), PE-CY5-conjugated rat anti-human CD49f 1:50 (BD Pharmingen, 551129, San Diego, California, USA), PE-conjugated mouse anti-human CD44 1:50 (BD Pharmingen, 555479, San Diego, California, USA), PE-conjugated mouse anti-human CD29 1:50 (BD Pharmingen, 556049, San Diego, California, USA), FITC-conjugated mouse anti-human CD24 1:50 (BD Pharmingen, 555427, San Diego, California, USA), and Biotinylated Thy-1 (Abcam, ab1154, Cambridge, MA, USA). Biotinylated antibodies were labeled by using Streptavidin-PE-CY5 (Invitrogen, SA1012, Carlsband, California, USA). MUC-1 antibody was conjugated by using FITC anti-mouse Igg1 (Biolegend, 406605, San Diego, California, USA). Isotype controls used were FITC mouse Igg1 (Biolegend, 400107, San Diego, California, USA), FITC mouse Igg2a (400207, Biolegend, San Diego, California, USA), PE mouse Igg2b (Biolegend, 401207, San Diego, California, USA), PE-CY5 mouse Igg1 (BD Bioscience, 550618, San Jose, California, USA), and PE-CY5 Rat Igg2a (Biolegend, 400509, San Diego, California, USA). Cells were stained at a cell concentration of 1 × 10^7^/ml for 30 min on ice followed by washes in Hanks' Balanced Salt Solution containing 2% fetal bovine serum (HBSS; Gibco BRL, Carlsband, California, USA). Flow-cytometry analysis and acquisition were performed by using the BD LSR II flow cytometer and BD FACSDIVA based software (BD Biosciences, San Jose, California, USA). Flow-cytometry sorting was performed by using FACSAria (BD Biosciences, San Jose, California, USA). The fluorescence expression level is arbitrarily designated as low (lo) if the log^10 ^of median fluorescence intensity (FI) is between about 0 to 2, medium (med), if the FI is between about 2.1 and 3.6, and high (hi) if the FI is higher than about 3.7.

### Immuno-FISH

Immuno-FISH was performed as previously described in [5].

## Results

### Intraductal transplantation as a model for human DCIS

**Figure 1:**
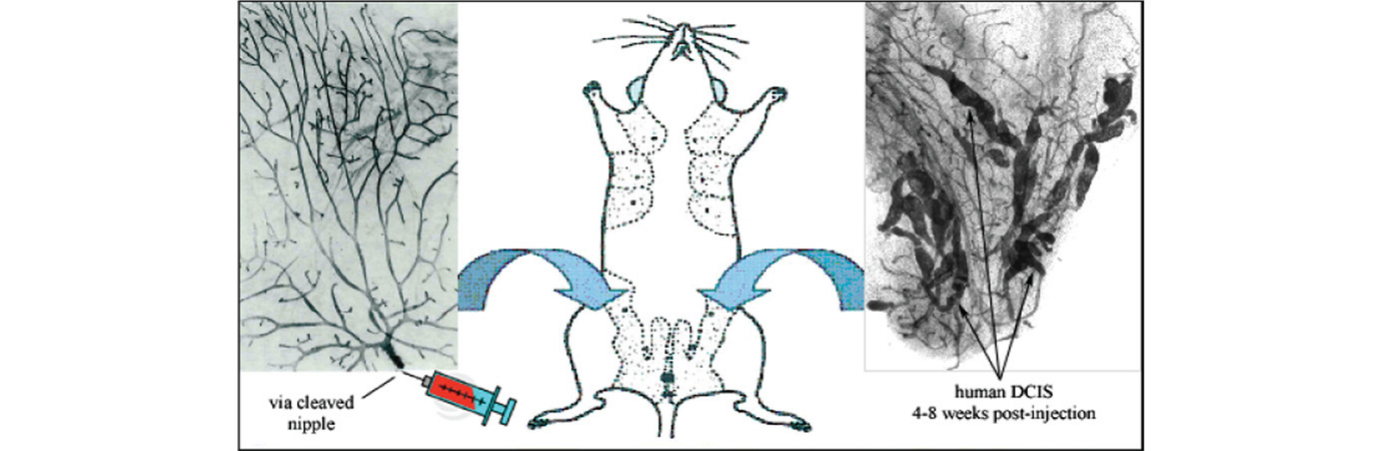
Intraductal HIM transplantation model mimics human breast cancer noninvasive-to-invasive progression. The model allows temporal analysis of many processes involved in early breast cancer invasive progression including intraductal cancer cell growth, the cell interactions with the surrounding normal epithelial and myoepithelial cells, and their escape into the surrounding stroma.

A limiting factor for studying human DCIS is the lack of suitable *in vivo *models. Furthermore, sufficient amounts of primary DCIS tissue available for research purposes are often inadequate. Therefore, the main goals were to design a method for expansion of human DCIS tissue *in vivo *and to develop models that would represent various subtypes of human DCIS. Many investigators have tried, with limited success, to develop xenograft models of human DCIS by a variety of methods including humanized fat-pad transplantation. The reason for the limited success may be that DCIS has been treated like an invasive lesion rather than a lesion limited in its growth potential under very restrictive conditions. With the idea that DCIS initiates and grows inside the ducts, we used intraductal transplantation. This approach involves injection of human DCIS cells directly into the primary ducts (Figure 1, and video [see Additional data file 1] demonstrate the intraductal-transplantation technique). We intentionally mimicked DCIS in the mouse to the closest condition to that found *in vivo *in the human disease. Two established DCIS cell lines, DCIS.COM, SUM-225, and a primary human DCIS lesion were used. The primary human DCIS cells were obtained by digestion of a primary human DCIS tumor. A pathologist diagnosed the primary human DCIS lesion. The researchers were blind to any other patient identifiers. The take rate was 90% for all three cell lines. The cells were injected at 40,000 cells in 2 μl. Three replicates for DCIS.COM (ratio of growth, 9:10, 5:6, and 6:6), two replicates for SUM-225 (ratio of growth, 5:6 and 5:6), and one replicate for the primary human DCIS, FSK-H7 (ratio of growth, 9:10) were found. None of the xenografts in this study was serially transplanted. Both DCIS.COM and SUM-225 are unique because they progress through a DCIS-like stage during tumor formation. The DCIS-like lesions generated by the DCIS.COM cell line slowly progressed to invasive lesions in 10 weeks, whereas those generated by the SUM-225 did not become invasive during the 10-week study period. In one study, three (25%) of 10 mammary fat pads injected with DCIS.COM generated invasive cancers in 10 weeks. This is in contrast to other established invasive breast cancer cell lines, such as MCF-7, that develop extensive invasive cancers in as early as 4 weeks after intraductal injections in six of six fat pads (unpublished data). None of the DCIS-like lesions generated by FSK-H7 (primary human DCIS cells) showed invasion during the 6-week study period.

**Figure 2:**
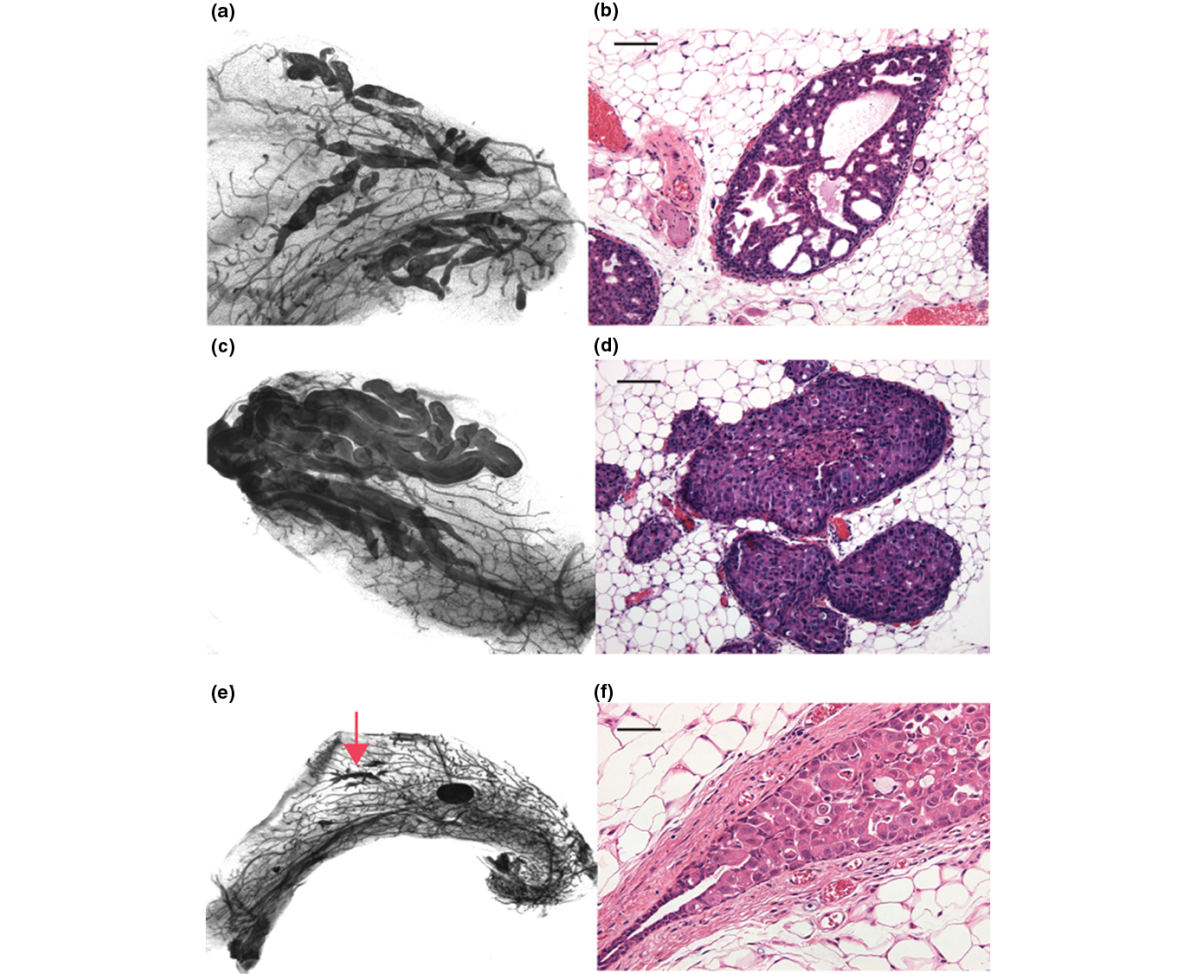
Hematoxylin and eosin (H&E) and whole-mount staining of intraductal xenografts. The panels show whole-mount and H&E staining of xenografts generated by intraductal transplantation of DCIS.COM **(a, b)**, SUM-225 **(c, d)**, and FSK-H7 **(e, f)**. The H&E figures depict ×20 magnification, and whole mounts were captured at ×1.6 (SUM-225 and DCIS.COM) and ×0.7 (FSK-H7). Cells were injected intraductally (20,000/μl cells in 2 μl of PBS) into the primary mammary ducts (*via *cleaved nipple) of intact 8-week-old immunocompromised SCID-beige female mice. The human primary DCIS was enzymatically digested into single cells before injections. The pictures were taken at 6 weeks after injection.

Figure 2 shows whole-mount and hematoxylin and eosin (H&E) stains of intraductal lesions generated from DCIS.COM (A, B), SUM-225 (C, D), and the primary human DCIS, FSK-H7 (E, F) 6 weeks after injection. SUM-225 exhibited a comedo pattern, whereas DCIS.COM exhibited a cribriform pattern. Previously, it was reported that DCIS.COM generates a comedo DCIS when injected subcutaneously [3]. This difference, most likely, is due to the subcutaneous *versus *intraductal sites of injections. Injection of the primary human DCIS cells (FSK-H7) generated apocrine-like DCIS lesions. Interestingly, the whole mounts show that many of the growths appear to be at the periphery and separated from the point of injection. This may be because the cells were injected when the SCID-beige mice were 6 to 8 weeks old, when the ducts are expanding. At the time of ductal expansion, the terminal ducts may provide a favorable environment for cancer cell growth. The reason for this effect is not known.

**Figure 3:**
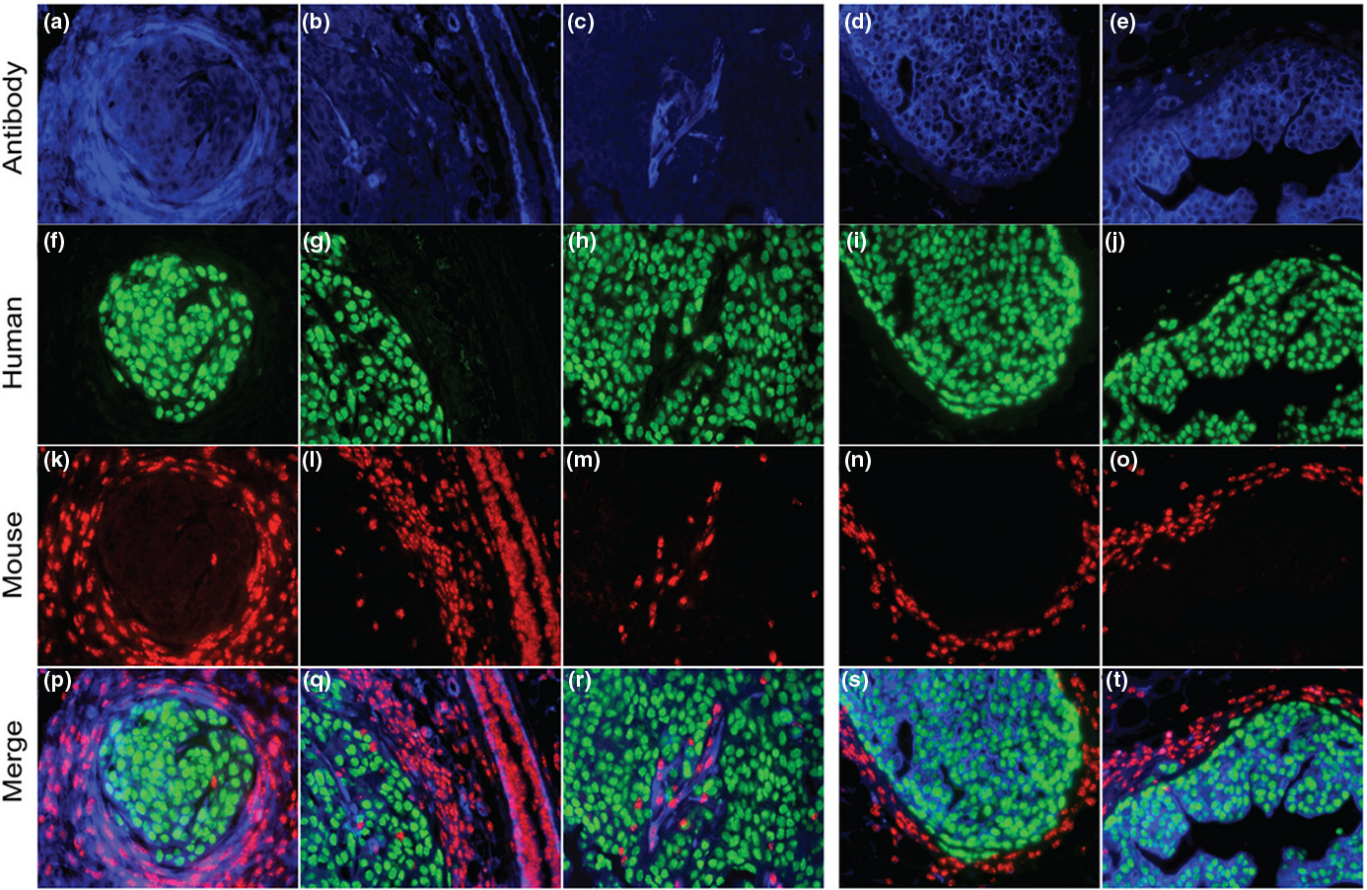
iFish analysis of a DCIS.COM intraductal xenograft. Human-derived DCIS.COM cells are contained within the boundaries of mouse myoepithelial cells and basement membrane. The panels depict immunofluorescence staining of paraffin sections from a fat pad containing DCIS.COM intraductal lesions by using anti-mouse smooth muscle actin antibody **(a-c)**, anti-human pan cytokeratin antibody **(d-e)**, iFish with fluorescently labeled human (green) **(f-j)**, and mouse (red) Cot-1 DNA as probes, and merged images of all panels **(p-t)**.

To assess the human and mouse origin of cells within the intraductal lesions, immuno-fish (FISH) was performed by using anti-mouse smooth muscle actin (SMA) (Figure 3A-C) and anti-human pan-cytokeratin antibodies (D-E), fluorescently labeled human (green) (F-J), and mouse (red) Cot1 DNA probes for FISH (K-O). The data show that human cells, indicated by green human Cot1 probe staining, are surrounded by mouse myoepithelial cells and myofibroblasts, indicated by a red mouse Cot1 probe. Therefore, the intraductal model recapitulates human DCIS by allowing the cancer cells to grow within the boundaries of the mouse basement membrane and myoepithelial cells. Furthermore, the model provides a unique opportunity for studying the factors that influence the process of cancer cell invasion into the stroma.

The transitional process of *in situ *breast lesion to invasive carcinoma is currently poorly understood. It has been proposed that the loss of myoepithelial cells concurrent with degradation of the basement membrane may play a major role in DCIS invasive progression. A recent report described the role of myoepithelial cells in this process in detail [5]. By subcutaneous transplantation of DCIS.COM, this group demonstrated that myoepithelial cells suppress, whereas fibroblasts enhance DCIS invasive progression. It was further demonstrated that the loss of myoepithelial expression of TGFβ R2 and SMAD-4, Gli-2, and MMP14 play a role in the invasive progression of DCIS [5]. Thus, the current model allows the study of many early processes of breast cancer cell invasion into the stroma, including the interactions of cancer cells with the surrounding normal luminal and myoepithelial cells as well as the basement membrane.

### Developing subtype-specific models of human DCIS xenograft lines

The long-term goal of our studies is to develop subtype-specific stable xenograft models of human DCIS by intraductal transplantation. The xenograft lines are termed stable if their morphology, subtype specificity, and invasive properties remain unchanged with repeated transplantation. Subtype-specific xenograft lines should allow one to study the distinct molecular and biologic mechanisms underlying the invasive progression of subtypes of human DCIS. We have identified two cell lines, DCIS.COM and SUM-225, that generate stable basal-like and Her-2-overexpressing DCIS-like lesions, respectively. Intraductal xenografts of a human primary DCIS, FSK-H7, generated Her-2-overexpressing lesions.

**Figure 4:**
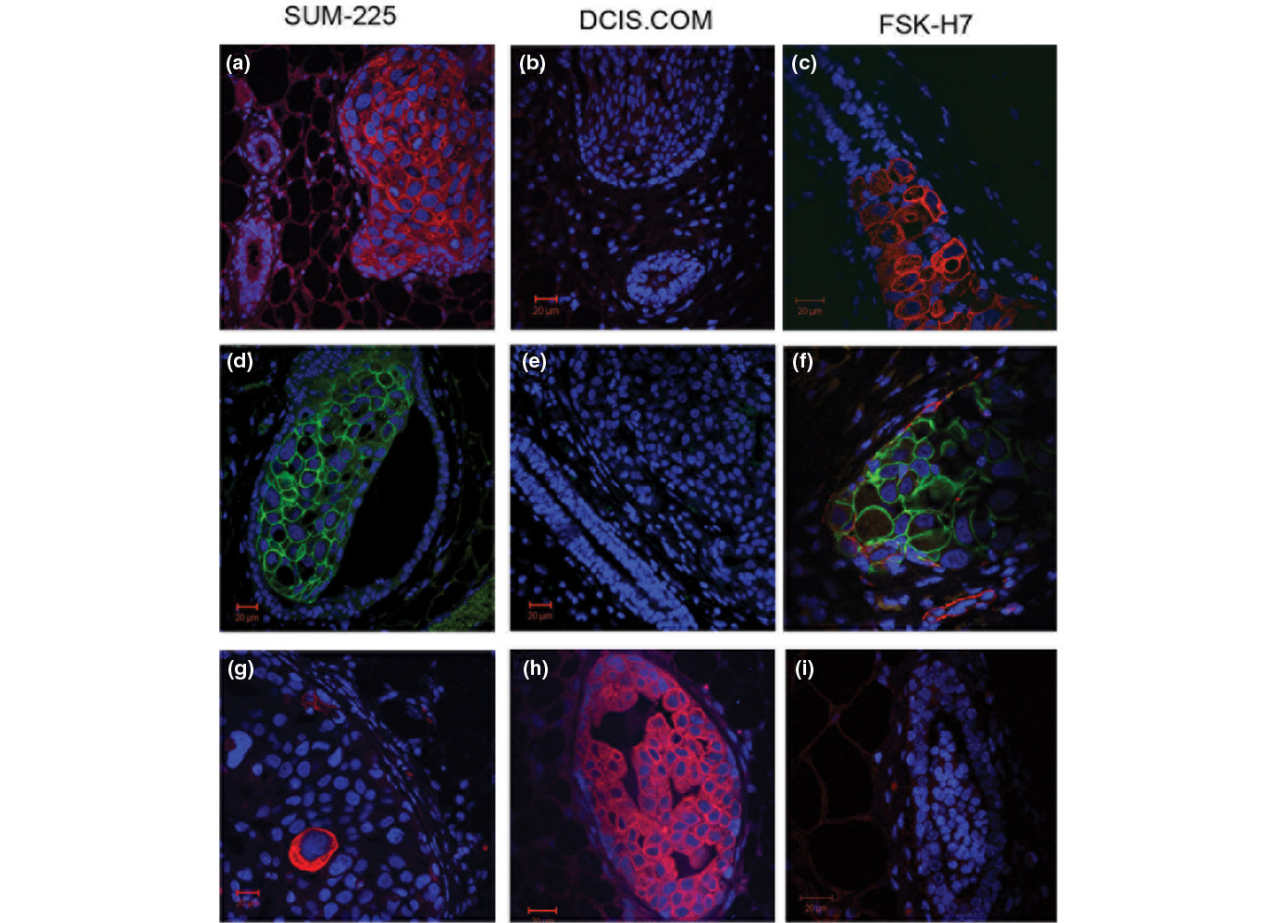
Expression of subtype-specific markers by the intraductally generated DCIS-like lesions. Intraductal lesions were generated by injection of SUM-225 **(a, d, g)**, DCIS.COM **(b, e, h)**, and FSK-H7 **(c, f, i)**. Six weeks after injection, fat pads containing intraductal lesions were removed and fixed, followed by paraffin embedding by using standard histological techniques. Paraffin sections were subjected to immunofluorescence studies with antibodies against human CK-19 **(a-c)**, Her-2 **(d-f)**, and CK-5 **(g-i)**. Primary antibodies were conjugated to secondary antibodies, Alexa-594 against mouse anti-human CK-5 and CK-19 (red), and Alexa-488 against rabbit anti-human Her-2 antibody (green).

Previous studies have shown that immunoassays may be used to predict the tumor subtypes identified with microarray analyses with a high degree of specificity [see Additional data file 3) [9,10]. Accordingly, immunofluorescence studies using anti-human cytokeratin-8 (CK-8), CK-19, CK-5, estrogen receptor (ER), Her-1, and Her-2 antibodies were performed. Figure 4 shows that SUM-225 and FSK-H7 lesions uniformly express CK-19 (A, C), whereas DCIS.COM lesions are CK-19 negative (B). SUM-225 and FSK-H7 express Her-2 (D-F), whereas CK-5 is exclusively expressed by DCIS.COM (G-L). All three intraductal lesions express CK-8; however, none of the lesions expresses ER (data not shown). These results suggest that DCIS.COM generates basal-type lesions, whereas SUM-225 and FSK-H7 generate Her-2-overexpressing lesions ([see Additional data file 1], and Figure 4).

### DCIS.COM and SUM-225 may contain distinct subpopulations with significantly higher *in vivo *growth potential

**Figure 5:**
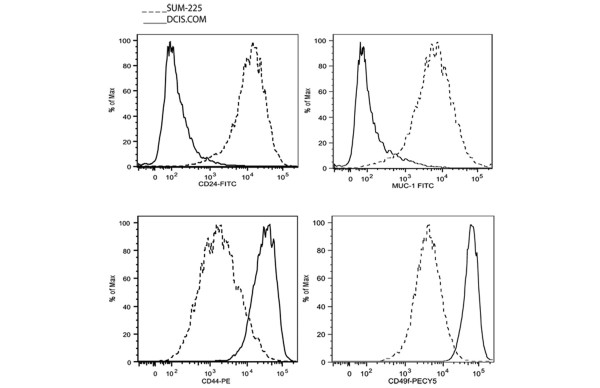
Flow-cytometry analysis of DCIS.COM and SUM-225. Cells grown in 2D were stained by using the indicated anti-human antibodies to the surface markers, CD44/CD24, CD49f/CD24, and CD49f/MUC-1. The antibodies were directly conjugated, except for MUC-1, which was conjugated by using an anti-mouse FITC antibody. The histograms show expression levels for the indicated surface markers. The expression level is arbitrarily designated as low (lo) if the log^10 ^of median fluorescence intensity (FI) is between about 0 and 2, medium (med) if the FI is between about 2.1 and 3.6, and high (hi) if the FI is higher than about 3.7.

We further used the intraductal model to test the hypothesis that xenografts representing two distinct subtypes of DCIS-like lesions may contain distinct tumor-initiating subpopulations. The two DCIS cell lines were used for this study. The cell lines were analyzed for the expression of human mammary basal and luminal surface markers. Figure 5 is a histogram depicting the relative expression level of the individual markers in DCIS.COM and SUM-225. The expression level is arbitrarily designated low (lo) if the log^10 ^of the median fluorescence intensity (FI) is between about 0 and 2, medium (med) if the FI is between about 2.1 and 3.6, and high (hi) if the FI is higher than about 3.7. The data in Figure 5 show that DCIS.COM expressed a hi level of the basal markers (CD44 and CD49f) and lo levels of the luminal markers (CD24 and MUC-1). In contrast, SUM-225 expressed medium levels of the basal markers and hi levels of the luminal markers. Various subpopulations expressing putative markers of mammary stem and progenitor cells were sorted with flow cytometry and transplanted. These subpopulations (Tables 1 and 2; column 2) were chosen based on previous reports of markers expressed by human breast stem/progenitors, mouse mammary epithelial stem cells, and human breast tumor-initiating cells [10,11]. The gates for the subpopulations sorted and transplanted are shown in the supplementary data [see Additional data file 4]. Fisher's Exact test was used to compare the *in vivo *growth potentials between the various subpopulations. Table 1 shows transplant results for two replicate experiments per sorted subpopulation per cell line. DCIS-like lesions are referred to as *in vivo *or intraductal growth. The number of fat pads containing positive intraductal growth over the total number of fat pads transplanted, growth fractions, and the total number of ducts containing intraductal growth are listed in Table 1, columns 3, 4, and 5, respectively. [See Additional data file 5.] Table 2 shows the results of Fisher's Exact test. As depicted in Table 1 in DCIS.COM, subpopulations expressing high levels of the basal markers and low levels of the luminal markers (CD49f^hi^/CD24^lo^, CD49f^hi^/MUC-1^lo^, and CD44^hi^/CD24^lo^) demonstrated higher *in vivo *growth fractions and generated more ducts containing lesions. However, as shown in Table 2 with Fisher's Exact test, none of the subpopulations in DCIS.COM achieved significantly higher growth fractions when compared with the random sort. Therefore, the majority of cells in DCIS.COM possessed tumor-initiating properties. In contrast, in SUM-225 cells, two subpopulations, CD49f^med^/CD24^hi ^and CD49f^med^MUC-1^hi^, achieved significantly higher growth fractions compared with the random sort (Table 2). These subpopulations also generated the largest number of ducts with lesions (Table 1). Because the tumor-initiating cells in DCIS.COM and SUM-225 cells expressed distinct surface markers, we postulate that the tumor-initiating cells may arise from distinct cell types. However, lineage-tracing experiments are required to establish whether different types of tumors arise from distinct cell types. Notably, although tumor formation was not an end point for this experiment, a few palpable tumors were found in the random sort as well as the CD44^hi^/CD24^lo ^groups in DCIS.COM. None of the SUM-225 subpopulations generated a tumor during the time course of our study (6 weeks).

**Table 1:** Transplant results

Cell line	Group	Number of positive fps/total	Ratio of positive growth	Total number of ducts positive	Number of tumors
DCIS.COM	Random sort	9/28	32%	22+	1
DCIS.COM	CD49f^hi^/MUC-1^med^	4/23	17%	11+	
DCIS.COM	CD49f^hi^/MUC-1^lo^	4/6	67%	9	
DCIS.COM	CD49f^med^/MUC-1^lo^	4/23	17%	8	
DCIS.COM	CD49f^med^/MUC-1^med^	4/6	67%	15	
DCIS.COM	CD49f^hi^/CD24^med^	3/8	38%	4	
DCIS.COM	CD49f^hi^/CD24^lo^	5/8	63%	18	
DCIS.COM	CD44^med^/CD24^med^	3/24	13%	3	
DCIS.COM	CD44^med^/CD24^lo^	1/6	17%	1	
DCIS.COM	CD44^hi^/CD24^lo^	7/24	29%	26	3
DCIS.COM	CD44^hi^/CD24^med^	3/6	50%	5	
SUM-225	Random sort	1/16	6%	7+	
SUM-225	CD49f^med^/MUC-1^hi^	6/11	55%	31+	
SUM-225	CD49f^med^/MUC-1^med^	0/6	0%	0	
SUM-225	CD49f^lo^/MUC-1^med^	1/12	8%	2	
SUM-225	CD49f^lo^/MUC-1^hi^	0/6	0%	0	
SUM-225	CD49f^med^/CD24^hi^	4/6	67%	22+	
SUM-225	CD49f^med^/CD24^med^	0/6	0%	0	
SUM-225	CD44^lo^/CD24^hi^	0/6	0%	0	
SUM-225	CD44^med^/CD24^med^	2/6	33%	8+	

A fixed number of cells (DCIS.COM = 5,000 cells in 2 μl and SUM-225 = 10,000 cells in 2 μl) sorted into different subpopulations or randomly sorted followed by injection into primary mammary ducts of SCID-beige female mice. Random sort refers to cells sorted based on forward and side scatter and irrespective of the expression of specific markers. Six weeks after intraductal injections, glands were removed, subjected to whole-mount staining, and the numbers of glands containing positive growth (DCIS-like lesions) were counted. The fraction of growth is referred to the number of fat pads containing positive DCIS-like lesions over the total number of fat pads transplanted. The columns contain the following data: Column 1 (Cell line): Cell line injected; Column 2 (Group): Subpopulations sorted and injected intraductally; Column 3 (Number of positive fps/total): Number of fat pads (fps or glands) containing positive growth (DCIS-like lesions) over the total number of fat pads (glands) transplanted; Column 4 (Ratio of positive growth): Fraction of fat pads containing positive growth (DCIS-like lesions); Column 5 (Total number ducts positive): Total number of positive ducts (ducts containing DCIS-like lesions) combined in all the positive glands per group (plus sign indicates one or more fat pads contained more than seven positive ducts); Column 6 (Number of tumors): Number of palpable tumors formed; these data were not included in the statistical evaluation. In DCIS.COM, the expression levels of the basal markers ranged from med to hi, and expression levels of luminal markers ranged from lo to med. Therefore, DCIS.COM does not contain CD49f^lo^-, CD44^lo^-, CD24^hi^-, or MUC-1^hi^-expressing cells. In SUM-225, the expression levels of basal markers CD49f and CD44 ranged from lo to medium, and the expression levels of the luminal markers CD24 and MUC-1 ranged from med to hi. Therefore, the groups do not include CD49f^hi^-, CD44^hi^-, CD24^lo^-, and MUC-1^lo^-expressing cells.

**Table 2:** Fisher's Exact test results

Comparisons		*P*
	
DCIS.COM		
Rand	CD49f^hi^CD24^med^	1.00
Rand	CD49f^hi^CD24^lo^	0.22
Rand	CD44^hi^CD24^med^	0.64
Rand	CD44^hi^CD24^lo^	1.00
Rand	CD44^med^CD24^med^	0.11
Rand	CD44^med^CD24^lo^	0.64
Rand	CD49f^hi^MUC-1^med^	0.34
Rand	CD49f^hi^MUC-1^lo^	0.17
Rand	CD49f^med^MUC-1^med^	0.17
Rand	CD49f^med^MUC-1^lo^	0.34

**SUM-225**		

**Rand**	**CD49f^med^CD24^hi^**	*0.01
Rand	CD49f^med^CD24^med^	1.00
Rand	CD44^med^CD24^med^	0.17
Rand	CD44^lo^CD24^hi^	1.00
**Rand**	**CD49f^med^MUC-1^hi^**	*0.01
Rand	CD49f^med^MUC-1^med^	1.00
Rand	CD49f^lo^MUC-1^hi^	1.00
Rand	CD49f^lo^MUC-1^med^	1.00

Pair-wise comparison of growth fractions between the various subpopulations and the random sort. Fisher's Exact test was used to compare the proportions of positive response (lesions in ducts) between subpopulations and the random sort (rand) in DCIS.COM and SUM-225. A *P *value < 0.05 indicates a statistically significant value.

## Discussion

As an attempt to study the various factors involved in breast cancer heterogeneity, beginning at the noninvasive stage, we developed an intraductal xenograft transplantation model. The model allows one to grow human breast noninvasive lesions and to follow their progression *in vivo*. This model offers several advantages over current subcutaneous-based models as well as some disadvantages. This mouse model closely mimics human DCIS because DCIS initiates inside the ducts, which provide a natural microenvironment for malignant growth. Furthermore, one can follow the steps involved in cancer cell growth intraductally, followed by invasion into the stroma. The interactions of cancer cells with their microenvironment, including other normal epithelial cells as well as components of the stroma, may then be studied at the molecular and cellular levels. Moreover, genetic manipulations of the microenvironment could potentially be used to study the roles of various factors involved in cancer cell invasion. One may block the interactions between cancer cells with their surrounding environment to halt or slow an invasive process. Furthermore, the diversity of the models will present the opportunity to investigate the effects of different chemoprevention agents on a broad spectrum of human DCIS, thus tailoring treatment to specific variants of DCIS.

A limitation of this model is that human cancer cells are transplanted into immunocompromised mouse mammary ducts, and thus, the interactions of mouse and human cells may be artificial. Furthermore, use of an immunocompromised host may mask the known important effects of some immune cells in the invasion process. This disadvantage is shared among all current human models of noninvasive breast cancers.

With the intraductal transplantation model, we tested the hypothesis that distinct subtypes of DCIS may contain distinct subpopulations of tumor-initiating cells that possess higher growth, invasion, and cancer stem cell potential. The distinct subpopulations enriched in tumor-initiating cells may ultimately determine a risk for DCIS malignant progression. Surface markers unique to normal human breast bi-potent and luminal progenitors, as well as human breast tumor-initiating cells, were chosen to isolate cells and to assess their *in vivo *growth and self-renewal potential. By using the two DCIS cell lines, our study shows that the subpopulations with higher growth potential may be distinct. The subpopulation with higher *in vivo *growth potential in the basal DCIS.COM cell line expressed CD44^hi^/CD24^lo^, CD49f^hi^/CD24^lo^, and CD49f^hi^/MUC-1^lo^. However, the difference did not achieve significance when compared with the random sort. This may be because our sample size was small or the majority of cells in DCIS.COM possess tumor-initiating properties or both. However, in the luminal-basal SUM-225 cell line, the CD49f^med^/CD24^hi ^and CD49f^med^/MUC-1^hi ^subpopulations possessed higher intraductal growth potential. CD24 and MUC-1 are both proposed luminal markers. Whether these cells possess luminal progenitor property will have to be established. The fact that the majority of cells in DCIS.COM were tumorigenic may explain why patients who have basal types of breast cancers do poorly compared with those with the other types of human breast cancers. Therefore, different types of tumors may contain different proportions of tumor-initiating cells or distinct subpopulations of tumor-initiating cells. Similar studies in mice show that different mouse mammary tumors may also contain different proportions of tumor-initiating cells (reviewed in [11]).

## Conclusions

We generated two distinct xenograft models of human breast noninvasive cancers by intraductal transplantation. This model offers a novel microenvironment for growth of human noninvasive lesions, mimics the diversity of human disease, and is a suitable tool with which to study the molecular and biologic mechanisms of breast noninvasive-to-invasive progression. Additional xenograft lines that represent other tumor subtypes, such as luminal A and luminal B, must be developed. Furthermore, xenograft lines from various types of primary noninvasive lesions should be developed. Molecular and cellular characterization of the temporal events leading to invasive progression unique to an individual patient's tumor type may offer new tailored preventive strategies and spare many patients from the development of invasive and metastatic breast cancers.

## Abbreviations

DCIS: ductal carcinoma *in situ*; DCIS.COM: MCF10DCIS.COM cell line; H&E: hematoxylin and eosin staining; HIM: human in mouse.

## Competing interests

The authors declare that they have no competing interests.

## Authors' contributions

FB designed experiments, interpreted and analyzed data, performed tissue culture, flow cytometry, and immunofluorescence, and prepared the manuscript. FSK designed experiments; introduced the idea of an intraductal transplant model for growth of DCIS cells. established xenograft lines by the intraductal method; performed tissue culture, H&E, and whole mounts; analyzed data; produced the video on the intraductal transplant model; and contributed to the preparation of the manuscript. HL designed experiments, performed flow cytometry and tissue culture, and contributed to the preparation of the manuscript. DE performed intraductal transplantation, immunohistochemistry, whole mounts, H&E, and captured microscopy images, and produced the video on the intraductal transplant model. SK performed tissue culture, flow cytometry, and immunofluorescence. JCH and EY performed tissue culture, flow cytometry, and immunofluorescence. PM and HWY performed statistical analysis of transplant data. DCA, JMR, and DM supervised the project, interpreted the results, and made substantive contributions to preparation of the manuscript. MH performed ImmunoFish. KP interpreted the results and made contributions to the preparation of the manuscript. All authors reviewed and approved the final version of the manuscript.

## Supplementary Material

Additional file 1 A Quicktime video demonstrating the intraductal transplantation technique. Click here for file

Additional file 2 A Word file listing primary antibodies. Click here for file

Additional file 3 A Word file containing classification of tumor subtypes by immunoassay. Expression of Her-2, ER, CK-5, and Her-1 by immunostaining may be used to predict tumor subtypes by microarray with a high degree of specificity [9,10]. Based on immunostaining, DCIS.COM generates basal, and SUM-225 and FSK-H7 generate Her-2-overexpressing DCIS-like lesions. Click here for file

Additional file 4 A TIF file containing flow-cytometry analysis of DCIS.COM and SUM-225 for co-expression of basal and luminal surface markers. SUM-225 and DCIS.COM were stained by using antibodies to CD44/CD24, CD49f/MUC-1, and CD49f/CD24. The gated subpopulations in each panel were sorted, and the *in vivo *growth potential was assessed by using intraductal transplantation. As illustrated by FACS-generated dot plots, the majority of cells in DCIS.COM are CD44^hi^, CD49f^hi^, MUC-1^med^, and CD24^med^. Therefore, DCIS.COM subpopulations (in Table 1) do not contain CD49f^lo^-, CD44^lo^-, CD24^hi^-, or MUC-1^hi^-expressing cells. SUM-225 cells were predominantly CD44^med^, CD49f^med^, MUC-1^hi^, and CD24^hi^. Therefore, SUM-225 subpopulations (in Table 1) do not include CD49f^hi^-, CD44^hi^-, CD24^lo^-, and MUC-1^lo^-expressing cells. Click here for file

Additional file 5 A Word file Subgroup comparisons by using Fisher's Exact test. Click here for file
