# Supplementary material for: An intraductal human-in-mouse transplantation model mimics the subtypes of ductal carcinoma in situ
Source: Breast Cancer Res. 2009 Sep 7;11(5):R66. doi: 10.1186/bcr2358 (PMC2790841; doi:10.1186/bcr2358)
Supplement: Additional file 2 — A Word file listing primary antibodies. [file bcr2358-S2.DOC]

Additional File 2. Primary Antibodies.

| **Antibody** | **Catalog Number** | **Company** |
| --- | --- | --- |
| ERα | NCL-ER-6F11 | Novocastra |
| SMA | 1A4-Dako | Dako |
| CK-5 | VP-C400 | Vector |
| CK-8 | 18-0185 | Zymed |
| Her-2 | RM-9103-SO | Labvision |
| CK-AE1/AE3 | M3515 | Dako |
| CK-19 | MS-198-P0 | Labvision |
| Her-1 | 28-0005 | Invitrogen |
| MUC-1 | 1423 | Stem Cell Technologies |
| CD10-APC | 340923 | BD Bioscience |
| EPCAM-FITC | 10109 | Stem Cell Technologies |
| AC133 Biotin | 130-090-664 | Miltenyi Biotec |
| CD49f-PECY5 | 551129 | BD Pharmingen |
| CD44-PE | 555479 | BD Pharmingen |
| CD29-PE | 556049 | BD Pharmingen |
| CD24-FITC | 555427 | BD Pharmingen |
| Thy-1 Biotin | ab1154 | Abcam |
