# Supplementary material for: An intraductal human-in-mouse transplantation model mimics the subtypes of ductal carcinoma in situ
Source: Breast Cancer Res. 2009 Sep 7;11(5):R66. doi: 10.1186/bcr2358 (PMC2790841; doi:10.1186/bcr2358)
Supplement: Additional file 3 — A Word file containing classification of tumor subtypes by immunoassay. Expression of Her-2, ER, CK-5, and Her-1 by immunostaining may be used to predict tumor subtypes by microarray with a high degree of specificity [9,10]. Based on immunostaining, DCIS.COM generates basal, and SUM-225 and FSK-H7 generate Her-2-overexpressing DCIS-like lesions. [file bcr2358-S3.DOC]

Additional File 3. Classification of tumor subtypes by immunoassay.

| **MA Profile** | **HER2** | **ER** | **CK-5** | **Her-1** |
| --- | --- | --- | --- | --- |
| Her-2 | + | - | - | +/- |
| Luminal A | - | + | - | - |
| Luminal B | + | + | - | - |
| Basal Like | - | - | +/- | +/- |

**SUM-225 and FSK-H7**

**DCIS.COM**
