# Supplementary figures and images for: An intraductal human-in-mouse transplantation model mimics the subtypes of ductal carcinoma in situ
Source: Breast Cancer Res. 2009 Sep 7;11(5):R66. doi: 10.1186/bcr2358 (PMC2790841; doi:10.1186/bcr2358)

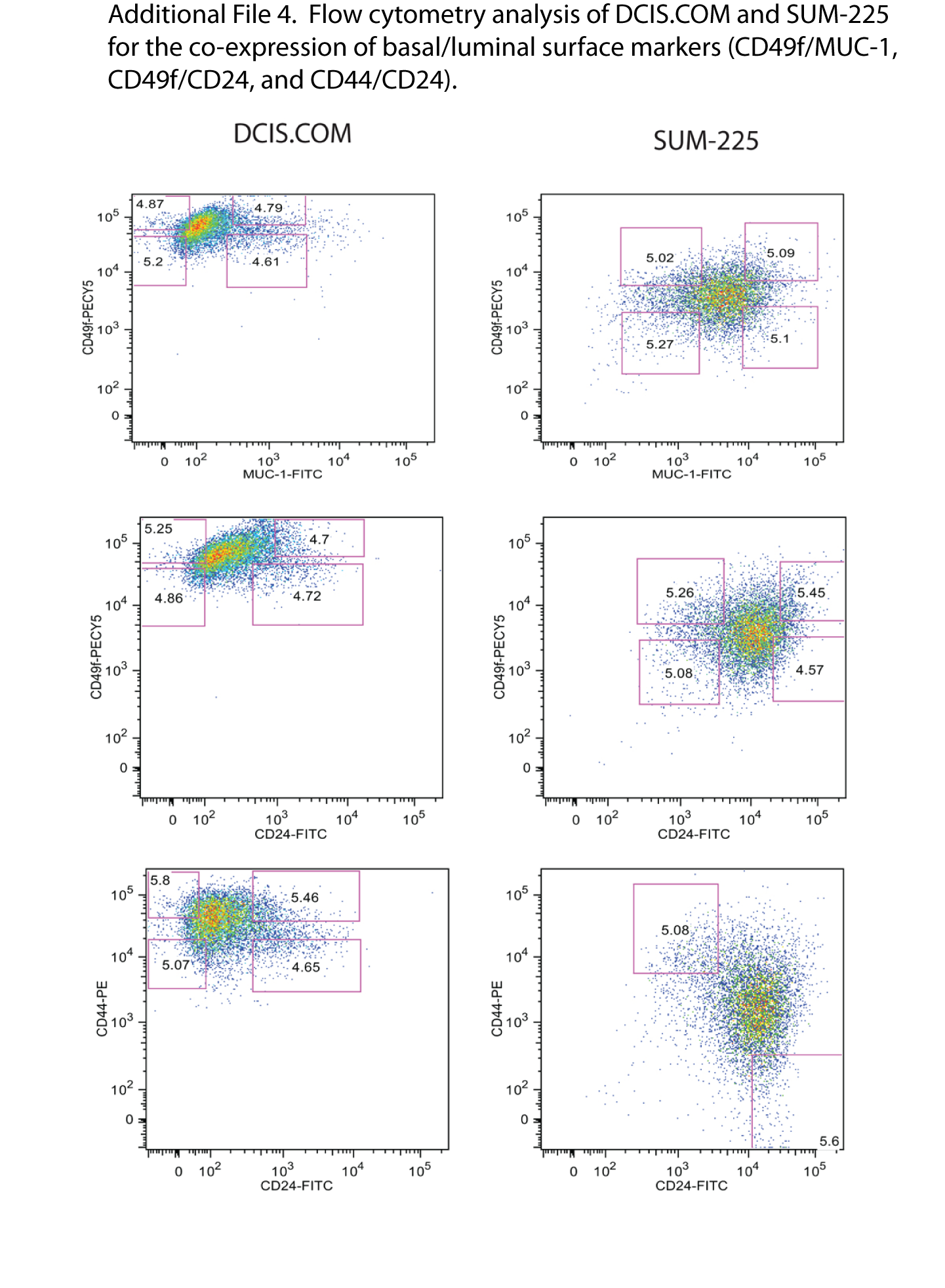

Supplement: Additional file 4 — A TIF file containing flow-cytometry analysis of DCIS.COM and SUM-225 for co-expression of basal and luminal surface markers. SUM-225 and DCIS.COM were stained by using antibodies to CD44/CD24, CD49f/MUC-1, and CD49f/CD24. The gated subpopulations in each panel were sorted, and the in vivo growth potential was assessed by using intraductal transplantation. As illustrated by FACS-generated dot plots, the majority of cells in DCIS.COM are CD44hi, CD49fhi, MUC-1med, and CD24med. Therefore, DCIS.COM subpopulations (in Table 1) do not contain CD49flo-, CD44lo-, CD24hi-, or MUC-1hi-expressing cells. SUM-225 cells were predominantly CD44med, CD49fmed, MUC-1hi, and CD24hi. Therefore, SUM-225 subpopulations (in Table 1) do not include CD49fhi-, CD44hi-, CD24lo-, and MUC-1lo-expressing cells. [file bcr2358-S4.TIFF]
