# Supplementary material for: An intraductal human-in-mouse transplantation model mimics the subtypes of ductal carcinoma in situ
Source: Breast Cancer Res. 2009 Sep 7;11(5):R66. doi: 10.1186/bcr2358 (PMC2790841; doi:10.1186/bcr2358)
Supplement: Additional file 5 — A Word file Subgroup comparisons by using Fisher's Exact test. [file bcr2358-S5.DOC]

**Additional File 5. Subgroup comparisons using Fisher’s exact test.**

Method

Fisher’s exact test was used to compare the proportions of positive response (lesions in ducts) between pairs of subpopulations. The highlighted rows indicate the groups that showed statistically significant differences in their growth potential.

Results

DCIS.COM

Among the 55 pair-wise comparisons in DCIS.COM subpopulations, the differences between the following pairs are significant at p=0.05:

| Comparison | | P |
| --- | --- | --- |
| Rand | CD49f^hi^CD24^med^ | 1.00 |
| Rand | CD49f^hi^CD24^lo^ | 0.22 |
| Rand | CD44^hi^CD24^med^ | 0.64 |
| Rand | CD44^hi^CD24^lo^ | 1.00 |
| Rand | CD44^med^CD24^med^ | 0.11 |
| Rand | CD44^med^CD24^lo^ | 0.64 |
| Rand | CD49f^hi^MUC-1^med^ | 0.34 |
| Rand | CD49f^hi^MUC-1^lo^ | 0.17 |
| Rand | CD49f^med^MUC-1^med^ | 0.17 |
| Rand | CD49f^med^MUC-1^lo^ | 0.34 |
| CD49f^hi^CD24^med^ | CD49f^hi^CD24^lo^ | 0.62 |
| CD49f^hi^CD24^med^ | CD44^hi^CD24^med^ | 1.00 |
| CD49f^hi^CD24^med^ | CD44^hi^CD24^lo^ | 1.00 |
| CD49f^hi^CD24^med^ | CD44^med^CD24^med^ | 0.15 |
| CD49f^hi^CD24^med^ | CD44^med^CD24^lo^ | 0.58 |
| CD49f^hi^CD24^med^ | CD49f^hi^MUC-1^med^ | 0.33 |
| CD49f^hi^CD24^med^ | CD49f^hi^MUC-1^med^ | 0.59 |
| CD49f^hi^CD24^med^ | CD49f^med^MUC-1^med^ | 0.59 |
| CD49f^hi^CD24^med^ | CD49f^med^MUC-1^lo^ | 0.33 |
| CD49f^hi^CD24^lo^ | CD44^hi^CD24^med^ | 1.00 |
| CD49f^hi^CD24^lo^ | CD44^hi^CD24^lo^ | 0.22 |
| CD49f^hi^CD24^lo^ | CD44^med^CD24^med^ | 0.01 |
| CD49f^hi^CD24^lo^ | CD44^med^CD24^lo^ | 0.14 |
| CD49f^hi^CD24^lo^ | CD49f^hi^MUC-1^med^ | 0.03 |
| CD49f^hi^CD24^lo^ | CD49f^hi^MUC-1^lo^ | 1.00 |
| CD49f^hi^CD24^lo^ | CD49f^med^MUC-1^med^ | 1.00 |
| CD49f^hi^CD24^lo^ | CD49^med^MUC-1^lo^ | 0.03 |
| CD44^hi^CD24^med^ | CD44^hi^CD24^lo^ | 0.64 |
| CD44^hi^CD24^med^ | CD44^med^CD24^med^ | 0.08 |
| CD44^hi^CD24^med^ | CD44^med^CD24^lo^ | 0.55 |
| CD44^hi^CD24^med^ | CD49f^hi^MUC-1^med^ | 0.13 |
| CD44^hi^CD24^med^ | CD49f^hi^MUC-1^lo^ | 1.00 |
| CD44^hi^CD24^med^ | CD49f^med^MUC-1^med^ | 1.00 |
| CD44^hi^CD24^med^ | CD49f^med^MUC-1^lo^ | 0.13 |
| CD44^hi^CD24^lo^ | CD44^med^CD24^med^ | 0.17 |
| CD44^hi^CD24^lo^ | CD44^med^CD24^lo^ | 0.64 |
| CD44^hi^CD24^lo^ | CD49f^hi^MUC-1^med^ | 0.32 |
| CD44^hi^CD24^lo^ | CD49f^hi^MUC-1^lo^ | 0.18 |
| CD44^hi^CD24^lo^ | CD49f^med^MUC-1^med^ | 0.18 |
| CD44^hi^CD24^lo^ | CD49f^med^MUC-1^lo^ | 0.32 |
| CD44^med^CD24^med^ | CD44^med^CD24^lo^ | 1.00 |
| CD44^med^CD24^med^ | CD49f^hi^MUC-1^med^ | 0.70 |
| CD49f^hi^MUC-1^lo^ | CD44^med^CD24^med^ | 0.02 |
| CD49f^med^MUC-1^med^ | CD44^med^CD24^med^ | 0.02 |
| CD44^med^CD24^med^ | CD49f^med^MUC-1^lo^ | 0.70 |
| CD44^med^CD24^lo^ | CD49f^hi^MUC-1^med^ | 1.00 |
| CD44^med^CD24^lo^ | CD49f^hi^MUC-1^lo^ | 0.24 |
| CD44^med^CD24^lo^ | CD49f^med^MUC-1^med^ | 0.24 |
| CD44^med^CD24^lo^ | CD49f^med^MUC-1^lo^ | 1.00 |
| CD49f^hi^MUC-1^lo^ | CD49f^hi^MUC-1^med^ | 0.03 |
| CD49f^med^MUC-1^med^ | CD49f^hi^MUC-1^med^ | 0.03 |
| CD49f^hi^MUC-1^med^ | CD49f^med^MUC-1^lo^ | 1.00 |
| CD49fhiMUC-1^lo^ | CD49f^med^MUC-1^med^ | 1.00 |
| CD49f^hi^MUC-1^lo^ | CD49f^med^MUC-1^lo^ | 0.03 |
| CD49f^med^MUC-1^med^ | CD49f^med^MUC-1^lo^ | 0.03 |

SUM-225

Among the 36 pair-wise comparisons in Sum 225 subpopulations, the differences between the following pairs are significant at 0.05 significance level:

| Comparison | | P |
| --- | --- | --- |
| Rand | CD49f^med^CD24^hi^ | 0.01 |
| Rand | CD49f^med^CD24^med^ | 1.00 |
| Rand | CD44^med^CD24^med^ | 0.17 |
| Rand | CD44^lo^CD24^hi^ | 1.00 |
| Rand | CD49f^med^MUC-1^hi^ | 0.01 |
| Rand | CD49f^med^MUC-1^med^ | 1.00 |
| Rand | CD49f^lo^MUC-1^hi^ | 1.00 |
| Rand | CD49f^lo^MUC-1^med^ | 1.00 |
| CD49f^med^CD24^hi^ | CD49f^med^CD24^med^ | 0.06 |
| CD49f^med^CD24^hi^ | CD44^med^CD24^med^ | 0.57 |
| CD49f^med^CD24^hi^ | CD44^lo^CD24^hi^ | 0.06 |
| CD49f^med^CD24^hi^ | CD49f^med^MUC-1^hi^ | 1.00 |
| CD49f^med^CD24^hi^ | CD49f^med^MUC-1^med^ | 0.06 |
| CD49f^med^CD24^hi^ | CD49f^lo^MUC-1^hi^ | 0.06 |
| CD49f^med^CD24^hi^ | CD49f^lo^MUC-1^med^ | 0.02 |
| CD49f^med^CD24^med^ | CD44^med^CD24^med^ | 0.45 |
| CD49f^med^CD24^med^ | CD44^lo^CD24^hi^ | 0.45 |
| CD49f^med^CD24^med^ | CD49f^med^MUC-1^hi^ | 0.04 |
| CD49f^med^CD24^med^ | CD49f^med^MUC-1^med^ | 0.04 |
| CD49f^med^CD24^med^ | CD49f^lo^MUC-1^hi^ | 0.04 |
| CD49f^med^CD24^med^ | CD49f^lo^MUC-1^med^ | 1.00 |
| CD44^med^CD24^med^ | CD44^lo^CD24^hi^ | 0.45 |
| CD44^med^CD24^med^ | CD49f^med^MUC-1^hi^ | 0.62 |
| CD44^med^CD24^med^ | CD49f^med^MUC-1^med^ | 0.45 |
| CD44^med^CD24^med^ | CD49f^lo^MUC-1^hi^ | 0.45 |
| CD44^med^CD24^med^ | CD49f^lo^MUC-1^med^ | 0.25 |
| CD44^lo^CD24^hi^ | CD49f^med^MUC-1^hi^ | 0.04 |
| CD44^lo^CD24^hi^ | CD49f^med^MUC-1^med^ | 0.04 |
| CD44^lo^CD24^hi^ | CD49f^lo^MUC-1^hi^ | 0.04 |
| CD44^lo^CD24^hi^ | CD49f^lo^MUC-1^med^ | 1.00 |
| CD49f^med^MUC-1^hi^ | CD49f^med^MUC-1^med^ | 0.04 |
| CD49f^med^MUC-1^hi^ | CD49f^lo^MUC-1^hi^ | 0.04 |
| CD49f^med^MUC-1^hi^ | CD49^lo^MUC-1^med^ | 0.03 |
| CD49f^med^MUC-1^med^ | CD49f^lo^MUC-1^hi^ | 0.03 |
| CD49f^med^MUC-1^med^ | CD49f^lo^MUC-1^med^ | 1.00 |
| CD49f^lo^MUC-1^hi^ | CD49f^lo^MUC-1^med^ | 1.00 |
